# Infection, Dysbiosis and Inflammation Interplay in the COVID Era in Children

**DOI:** 10.3390/ijms241310874

**Published:** 2023-06-29

**Authors:** Laura Marinela Ailioaie, Constantin Ailioaie, Gerhard Litscher

**Affiliations:** 1 Department of Medical Physics, Alexandru Ioan Cuza University, 11 Carol I Boulevard, 700506 Iasi, Romania; lauraailioaie@yahoo.com (L.M.A.); laserail_mail@yahoo.com (C.A.); 2 President of the International Society for Medical Laser Applications (ISLA Transcontinental), German Vice President of the German–Chinese Research Foundation (DCFG) for TCM, Honorary President of the European Federation of Acupuncture and Moxibustion Societies, 8053 Graz, Austria

**Keywords:** autoantibodies, ACE-2, hyperinflammation, immune imbalance, gut, long COVID, microbiome, mimicry, permanent tissue damage, post-infectious, viral persistence

## Abstract

For over three years, severe acute respiratory syndrome coronavirus 2 (SARS-CoV-2) in children and adolescents has generated repercussions, especially a few weeks after infection, for symptomatic patients who tested positive, for asymptomatic ones, or even just the contacts of an infected person, and evolved from severe forms such as multisystem inflammatory syndrome in children (MIS-C) to multifarious clinical manifestations in long COVID (LC). Referred to under the umbrella term LC, the onset of persistent and highly heterogeneous symptoms such as fatigue, post-exertion malaise, cognitive dysfunction, and others have a major impact on the child’s daily quality of life for months. The first aim of this review was to highlight the circumstances of the pathophysiological changes produced by COVID-19 in children and to better understand the hyperinflammation in COVID-19 and how MIS-C, as a life-threatening condition, could have been avoided in some patients. Another goal was to better identify the interplay between infection, dysbiosis, and inflammation at a molecular and cellular level, to better guide scientists, physicians, and pediatricians to advance new lines of medical action to avoid the post-acute sequelae of SARS-CoV-2 infection. The third objective was to identify symptoms and their connection to molecular pathways to recognize LC more easily. The fourth purpose was to connect the triggering factors of LC with related sequelae following acute SARS-CoV-2 injuries to systems and organs, the persistence of the virus, and some of its components in hidden reservoirs, including the gut and the central nervous system. The reactivation of other latent infectious agents in the host’s immune environments, the interaction of this virus with the microbiome, immune hyperactivation, and autoimmunity generated by molecular mimicry between viral agents and host proteins, could initiate a targeted and individualized management. New high-tech solutions, molecules, probiotics, and others should be discovered to innovatively solve the interplay between RNA persistent viruses, microbiota, and our immune system.

## 1. Introduction

For the past four years, humanity has lived under the sign of uncertainty, infectivity, and mortality generated by the severe acute respiratory syndrome coronavirus 2 (SARS-CoV-2) that first appeared in Wuhan, China, in late 2019 and generated the coronavirus disease 2019 (COVID-19), responsible for the COVID-19 pandemic. Along with COVID-19 (2020 to present), there have been previous outbreaks that have been classified as public health emergencies of international concern by the World Health Organization (WHO), whose stimuli for the adoption of the 2005 International Health Regulations (IHR) as a treaty with 196 states were the following: the 1999–2000 influenza outbreak in Israel, the 2003 severe acute respiratory syndrome (SARS) pandemic, the 2009 H1N1 influenza pandemic, the 2014 Ebola outbreak, the 2015 Middle East respiratory syndrome (MERS) outbreak, poliomyelitis (2014 to present) [1,2,3].

Assumed as a relative of SARS and MERS, the SARS-CoV-2 betacoronavirus set the COVID-19 pandemic in motion by infecting primarily the lower respiratory tract and manifesting as pneumonia in humans. Also popularly known as the Wuhan seafood market pneumonia coronavirus, it is a positive-sense single-stranded ribonucleic acid (RNA) virus that infected the first symptomatic patients on 8 December 2019. WHO identified the first suspected cases three weeks later by genomic sequencing after RNA testing on samples taken from a patient with pneumonia during the initial outbreak in Wuhan. On 30 January 2020, the WHO declared this disease a Public Health Emergency of International Concern and characterized the outbreak as a pandemic on 11 March 2020. In the last century, and especially in the last three decades, an extensive percentage of viral outbreaks have been caused by RNA viruses, such as influenza, dengue, Zika, Ebola, and so on. Historically, unimaginable features have been discovered about the core replication machinery put into action by coronaviruses as a paradox among positive-strand RNA viruses because they use both a processivity factor and possess proofreading activity that tends to resemble deoxyribonucleic acid (DNA) organisms, in addition to the constitutive proteins involved in prompt RNA synthesis, often exploited by negative-strand RNA viruses [4,5,6,7,8,9].

The impact of COVID-19 on public health was considered severe, with the potential for community and international spread, so to reduce transmission, countries have put in place “stay at home” orders, strict lockdowns, and restrictions on public liberties. Although in children the infection usually resulted in mild illness, there has also been the rare complication of multisystem inflammatory syndrome in children (MIS-C) requiring emergency services among pediatric patients to save their lives [10].

The 2005 IHR is a valuable instrument of international law that is legally binding on many countries to build the capacity to identify and report potential public health emergencies worldwide. Investing WHO with more official authority and a proper understanding of the potential of IHR will strengthen global health security—a huge investment in human and animal health—while mitigating the possible extensive economic effects of the next global health emergency. The actual extent and trend of emergency department (ED) utilization, particularly in pediatrics, depends on both the epidemic disease and the mandated public health measures and how these affect decision-making, so policy makers need to be aware of the fact that fear of the virus in the general population certainly affects the response to public health advice, particularly in children, where there is a great inequity in considering the impact of the epidemic, which needs to be addressed in the future [1,10,11].

Many driving factors such as globalization, increased trade, urbanization, changing human behavior, and increased prevalence of viral diseases among animals can generate such pandemics as COVID-19, demonstrating that under the above-mentioned circumstances, viral diseases could easily spread between nations, having an impact on their economic stability. The COVID-19 pandemic has had an important global mortality rate and has presented a truly new challenge for medical systems worldwide to control this event quickly and correctly with multiple unforeseen consequences and harmful repercussions. It led to a special socioeconomic break due to the strict protection measures adopted by the authorities. The latest novel pandemic virus, i.e., SARS-CoV-2, has been shown to differ from the other two previous human coronaviruses related to genome structure, site of infection, transmissibility, and other important characteristics, all of which are implicated in its different lethal pattern. Like other RNA viruses, SARS-CoV-2, while adapting to its new human hosts, was susceptible to genetic evolution with the development of mutations over time, resulting in mutant variants, some of which were considered variants of concern by WHO, with an important impact on global public health. Many elderly or chronically ill people lost their lives, but also young people and children through the so-called “cytokine storm” (CS), an abnormal response of the host’s immune system (IS), characterized by an excessive discharge of proinflammatory cytokines/chemokines, which is activated by the SARS-CoV-2 virus with all its multiple and unpredictable clinical manifestations, and which could not be effectively managed with the drugs available at the beginning. Molecular mimicry allows this virus to affect the self-tolerance of the host’s IS, and the efforts of IS to eliminate SARS-CoV-2 can trigger autoimmunity by hyperactivating both the innate and adaptive ISs. This IS hyperactivity and production of autoantibodies after COVID-19 can trigger autoimmune diseases [12,13,14,15,16,17,18].

The first aim of this review was to highlight the circumstances of the pathophysiological changes produced by COVID-19 in children and to better understand hyperinflammation in COVID-19 and how MIS-C, as a life-threatening condition, could have been avoided in some patients. Another goal was to better identify the interplay between infection, dysbiosis, and inflammation at a molecular and cellular level, to better guide scientists, physicians, and pediatricians to advance new lines of medical action to avoid the post-acute sequelae of SARS-CoV-2 infection (PASC). Referred to under the umbrella term long COVID (LC), the onset of persistent and highly heterogeneous symptoms such as fatigue, post-exertion malaise, cognitive dysfunction, and others have a major impact on the child’s daily quality of life for months. The third objective was to identify symptoms and their connection to molecular pathways to recognize LC more easily. The fourth purpose was to connect the triggering factors of LC with related sequelae following acute SARS-CoV-2 injuries to systems and organs, the persistence of the virus, and some of its components in hidden reservoirs, including the gut and the central nervous system. The reactivation of other latent infectious agents in the host’s immune environments, the interaction of this virus with the microbiome, immune hyperactivation, and autoimmunity generated by molecular mimicry between viral agents and host proteins, could initiate a targeted and individualized management.

Worldwide, as of 12 April 2023, there were 762,791,152 confirmed cases of COVID-19, including 6,897,025 deaths, reported to WHO. As of 10 April 2023, a total of 13,340,275,493 vaccine doses have been administered [19].

As of May 2020, the US Centers for Disease Control and Prevention (CDC) has tracked case reports of MIS-C, the rare but life-threatening condition in children associated with COVID-19, with a total of 9445 patients with MIS-C and 78 deaths, as the last updated cases reported to CDC before 31 March 2023. More than half of these MIS-C patients were aged between 5 and 13 years, with a mean of 9 years. In total, 98% of the cases had a positive test result for SARS-CoV-2, and the difference of 2% were contacts of confirmed persons with COVID-19; 57% of children were Hispanic/Latino (2351 patients) or Black, non-Hispanic (2709 patients); of all reported patients with MIS-C, 60% were male [20].

Except for neonates, initial data showed that SARS-CoV-2 infection in children and young people (CYP) or teenagers is less severe; however, some patients reported LC, but the most feared complication was the post-severe infectious disease occurring a few weeks after the onset of a mild or asymptomatic form of COVID-19 or after contact with a sick person, which has been identified as a multisystemic hyperinflammatory reaction, i.e., MIS-C, and continued international scientific cooperation is still needed to establish the full impact, especially from a neurological perspective [21,22,23,24].

Understanding the symptoms, infectivity, and transmission patterns of SARS-CoV-2 in CYP has been critical to enable, develop, and improve control measures for COVID-19 in all ages. MIS-C and multisystem inflammatory syndrome in neonates (MIS-N) post-COVID involve an internationally acknowledged consensus definition and international data complete series to make better the management and to plan clinical trials and future targeted therapies to prevent and control clinical sequelae. In low-resource settings, screening for COVID-19 in CYP is a useful way to risk-stratify most patients presenting to EDs [25,26,27].

Early clinical investigations in the spring of 2020 revealed that children and adolescents presented with mild symptoms, especially dry cough, associated or not with fever, fatigue, nasal congestion, gastrointestinal (GI) symptoms, and others, but with symptoms different from those of adult patients. Since there were also many asymptomatic infections, special protective measures had to be taken. Some children who had mild forms of the disease, who were asymptomatic, or had previously been in contact with people sick with COVID-19 developed a form of severe systemic inflammatory disease about a month later after the initial infection or contact that could have led to multiple organ failure and required emergency hospitalization, especially in intensive care, to save their lives. In the first instance, through disparate case reports, the use of different case definitions and even distinct names in different countries or continents (later called Pediatric Multisystem Inflammatory Syndrome Temporarily Associated with SARS-CoV-2 (PIMS-TS) in Europe, and MIS-C in the USA), the new pediatric inflammatory syndrome with multisystem involvement, described in association with SARS-CoV-2, did not initially provide sufficient insight into the full clinical spectrum, epidemiological and immunological features, as well as prognosis, having a heterogeneous spectrum. However, despite the severe initial presentation, the overall short-term outcome was reported initially to be favorable for most cases, with few recorded deaths [28,29,30].

MIS-C phenotypes across the COVID-19 pandemic proved to be severe with an unpredictable course and a substantial risk of cardiogenic shock. MIS-C patients in a later stage of the pandemic displayed a more severe phenotype, reflecting the impact of distinct SARS-CoV-2 variants [31].

Most patients, although negative for SARS-CoV-2 at presentation, had immunoglobulin type M (IgM) or immunoglobulin type G (IgG) antibodies, or both, indicating a prior contamination with this virus. Although the children presented with a wide spectrum of signs and symptoms and disease severity, ranging from fever and inflammation to myocardial injury, shock, and the development of coronary aneurysms, comparison with Kawasaki disease (KD) and KD shock syndrome certified a clear discrimination from all other well-known pediatric inflammatory diseases [32].

There has been limited insight into the viral antibody fingerprint following SARS-CoV-2 infection in children. An extensive immunoprofiling of the SARS-CoV-2 proteome in patients with mild/moderate or severe COVID-19 versus MIS-C and compared to hospitalized controls delineated distinctive cytokine responses, (IgM)/(IgG)/immunoglobulin type A (IgA) epitope diversity, antibody binding and avidity. Binding kinetics of antibodies to 24 SARS-CoV-2 proteins highlighted antibody features that differentiate between children with mild/moderate versus severe COVID-19 or MIS-C. Therefore, highly important SARS-CoV-2 antibody signatures in children associated with disease severity could be used to define virus neutralization targets and to design serodiagnostic tests, prognostic algorithms, therapies, and vaccines in these significant but insufficiently studied patients [33].

Although it is not evident why some children develop MIS-C, it has been determined that patients diagnosed with this condition produced superior titers of IgA antibodies and top functional IgG antibodies, which could indicate local inflammation of the GI mucosa, potentially induced by an unremitting SARS-CoV-2 intestinal infection that triggered an uninterrupted discharge of SARS-CoV-2 antigens [34].

## 2. Long COVID Impact on the Health of Children and Adolescents

### 2.1. General Data

At the beginning of the pandemic triggered by SARS-CoV-2, both children and adolescents were estimated to have a low incidence of infection, low risk of hospitalization, and modest mortality rates compared to adults. The explanation for this situation was later elucidated by the fact that the epidemiology of COVID-19 among children and adolescents was difficult to assess, especially since in these age groups there was an increased prevalence of asymptomatic infections and testing was lower than in grownups. Subsequent studies revealed that the incidence rates of SARS-CoV-2 infection in the pediatric population were similar to those in adults, but many cases have evolved asymptomatically or with less severe clinical signs [35,36,37,38].

**Figure 1:**
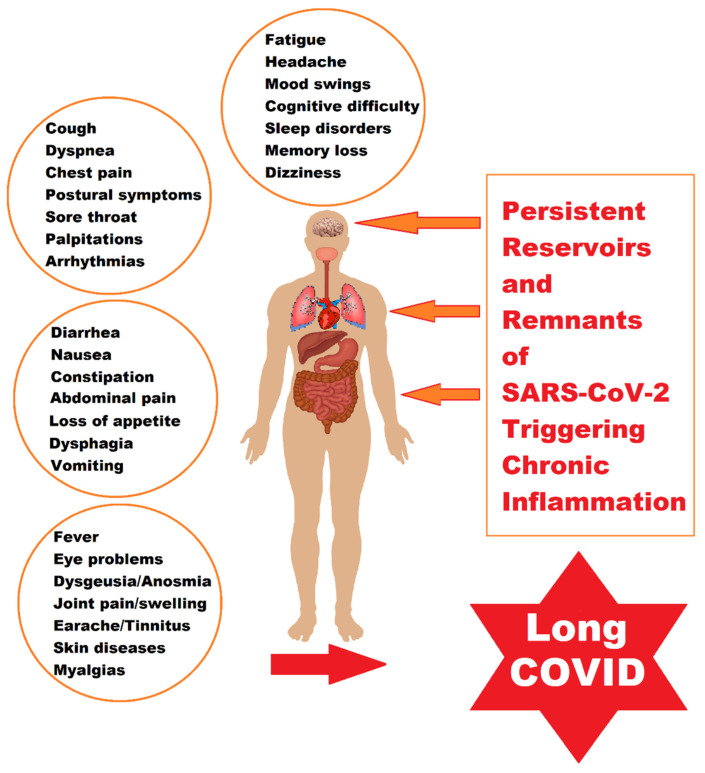
Clinical manifestations of LC in children and adolescents (Figure 1 was imagined and drawn by L.M.A. using Microsoft Paint 3D for Windows 10 and using completely free picture material from SeekPNG.com (accessed on 20 May 2023), for which we are very grateful).

After SARS-CoV-2 infection, a variable percentage of patients still present a variety of clinical signs or even new symptoms, which reappear or continue to persist after the initial infection. Chronic symptoms after COVID-19 called PASC or LC can affect up to 30% of all infected people. These symptoms may continue to manifest for longer periods of time. Although most children and adolescents recover from COVID-19, some of them may have symptoms that persist for weeks or months after SARS-CoV-2 infection. The proportion of children who subsequently develop a post-COVID-19 condition varies widely. The frequency of post-COVID-19 symptoms is related to age; the older children and adolescents are, the greater the risk of these manifestations, but the risk increases especially in those who have had a severe acute form of COVID-19. Clinical manifestations usually described in children include poor concentration, fatigue, nausea, headache, muscle and joint pain, cough, diarrhea, and fever, but many systems or organs may be involved, so some patients may describe multiple symptoms, as shown in Figure 1. Clinical manifestations may recur over time after initial recovery from an acute episode of COVID-19 or may fluctuate or recur over time. It usually resolves within 1–5 months, but some studies report that post-COVID-19 symptoms persist for much longer periods, so further research is needed to clarify more aspects of LC both in adults and in the pediatric population [39,40,41].

Persistent symptoms after COVID-19 were cataloged in May 2020 by patients on Twitter and other social networks under the name “long COVID”. This term was first used by Dr. Elisa Perego in conversations with her patients. Other names for this new pathology are post-COVID syndrome or post-COVID conditions, late sequelae of COVID-19, long-haul COVID, post-acute COVID-19 or post-COVID-19, long-term COVID-19, long-term effects of COVID, chronic COVID, chronic COVID-19 syndrome (CCS), and PASC, the latter name being more commonly used in the United States (US) [42,43,44].

The National Institutes of Health (NIH), as the main agency of the US government responsible for biomedical and public health research, following a virtual workshop organized in collaboration with the National Institute of Allergy and Infectious Diseases, on 3–4 December 2020, defined post-acute manifestations of COVID-19 as persistent symptoms and/or delayed or long-term complications of SARS-CoV-2 infection more than 4 weeks after the onset of the disease [45,46,47].

WHO convened an international panel of experts from within the organization, together with clinicians, researchers, and 265 patients, to find a consensus on the post-COVID-19 clinical case definition. Using the Delphi methodology, i.e., “a structured communication technique originally developed as a systematic, interactive forecasting method that relies on a panel of experts”, they published on 6 October 2021 the clinical case definition of LC, which includes 12 domains and 88 words. This definition refers to adults only and states that: “post-COVID-19 condition occurs in individuals with a history of probable or confirmed SARS-CoV-2 infection, usually 3 months from the onset of COVID-19, with symptoms that last for at least 2 months and cannot be explained by an alternative diagnosis. Common symptoms include fatigue, shortness of breath, cognitive dysfunction but also others, which generally have an impact on everyday functioning. Symptoms may be new onset, following initial recovery from an acute COVID-19 episode, or persist from the initial illness. Symptoms may also fluctuate or relapse over time”. A separate definition may be applicable for children [48,49,50].

The following year, on 2 February 2022, the UK’s National Institute for Health and Care Excellence (NICE) published guidelines on the characteristics of this new “long COVID” pathology that emerged after acute COVID-19. The clinical manifestations were listed and included two subdomains by time frame: ongoing symptomatic COVID-19, defined as signs and symptoms of COVID-19 from 4 to 12 weeks, and post-COVID-19 syndrome, defined as signs and symptoms that develop during or after an infection consistent with COVID-19 and continue for more than 12 weeks [51,52,53].

If most studies on the definition of clinical case condition for long-term COVID have focused mainly on the pathology of adults, for children and adolescents there was no concrete definition from international agencies or WHO experts. There was an urgent need to increase the interest of clinicians, researchers, patients, and their families regarding the risk of these complications after acute COVID-19 for a better understanding of the pathophysiology, reporting, appropriate management, and impact on the health of children as future adults. That is why Stephenson et al. from the Institute of Child Health Population, Policy and Practice, as well as other child health institutions in London, UK, undertook a search for the definition of “long Covid (post-COVID-19 condition)” in CYP, using a Delphi process of online surveys and a virtual consensus meeting, in three phases. In total, 120 people with relevant expertise in service delivery, research (or a combination of research and service delivery), and lived experience of CYP (aged between 11 and 17 years), participated. The recommended clinical case definition for long-term COVID was the Delphi process modified after the WHO and proposed as follows: “post-COVID-19 condition occurring in young people with a history of confirmed SARS-CoV-2 infection, with at least one persistent physical symptom for at least 12 weeks after initial testing that cannot be explained by an alternative diagnosis. Symptoms affect daily functioning, may continue or develop after infection with COVID, and may fluctuate or recur over time. The positive COVID-19 test referred to in this definition may be a lateral flow antigen test, a PCR test, or an antibodies test”. The authors argued that expanded acceptance of this definition could be beneficial in assessing the prevalence, course, and long-term outcome of COVID in CYP [54].

In a very short time, WHO convened 27 experts who participated in a virtual meeting on 13 September 2022 to develop a clinical case definition for the post-COVID-19 condition in children and adolescents. Based on the best available evidence and by general consensus, experts have accepted the case definition for post-COVID-19 in children and adolescents, which was published on 16 February 2023. It states that the condition occurs in “people with a history of confirmed or probable SARS-CoV-2 infection, when experiencing for at least 2 months symptoms that initially appeared within 3 months of acute COVID-19”. Chronic fatigue syndrome, headache, altered taste/smell, concentration difficulties, and anxiety were cited as common post-COVID-19 symptoms in children and adolescents. The persistence of three or more individual symptoms after acute SARS-CoV-2 infection may be helpful in identifying these patients. Regarding the very diverse symptomatology, it was stated that “symptoms generally influence daily functioning such as changes in eating habits, physical activity, behavior, school performance, social functions (interactions) with friends, peers, family) and developmental stages”. At the same time, the WHO noted that “symptoms may have a new onset after initial recovery from an acute episode of COVID-19 or may persist after the initial illness. They may also fluctuate or recur over time”. The experts concluded that post-COVID-19 status in children and adolescents is a highly dynamic field of investigation, with significant heterogeneity in presentations. As the symptoms evoked by patients or their family members are common and can be found together with others in childhood infections and diseases, the WHO proposes a broad list of potential manifestations attributed to post-COVID-19 that should be kept in mind [55,56].

A surprising long-term feature of post-COVID-19 is that it can affect adults who have survived COVID-19, regardless of the severity of the disease, and even children who have had an asymptomatic form of COVID-19, presenting with a state of chronic fatigue, headache, muscle pain, chest pain, palpitations, or cognitive dysfunction, and persisting for at least 6 months [57,58].

### 2.2. Clinical Studies on Long COVID in the Pediatric Population

The first LC cases in children were published by Ludvigsson in Sweden, who was contacted initially in October 2020 on social media by the parents of three children who had COVID-19 and were left with persistent symptoms. All five cases published subsequently by the author were described as manifesting a state of fatigue, dyspnea, and palpitations or chest pain, occurring 2 months after the onset of COVID-19. Only one child was known to have allergic asthma and mild autistic disorder before developing COVID-19. The parents reported that all three children also had abdominal pain, memory loss, depression, skin rashes, and muscle pain. In addition, four of the five children complained of headaches, difficulty concentrating, muscle weakness, dizziness, and sore throats. Other symptoms, such as recurrent fever, sleep disorders, joint pain, diarrhea, and vomiting, were reported by one child each; after 2 months, loss of smell and taste, decreased appetite, chronic cough, and numbness were observed. The children’s symptoms improved after 6–8 months, but all remained with chronic fatigue, and none were able to return to full-time school classes. Children may have similar LC manifestations as adults, and females may be more affected [59].

Another study published at the beginning of the COVID-19 pandemic was conducted by Buonsenso et al. on 129 children diagnosed with COVID-19 between March and November 2020, of which 6 children with severe neurocognitive deficiency were excluded. After acute COVID-19, three developed MIS-C, and two developed myocarditis. Symptoms present more than 60 days after initial diagnosis included: insomnia, respiratory symptoms (including chest pain and chest tightness), nasal congestion, fatigue, muscle and joint pain, and difficulty concentrating. An important and unexpected aspect was that the children with asymptomatic or paucisymptomatic COVID-19 also presented chronic symptoms after diagnosis. There is a need for pediatricians, health experts, and policy makers to take action to reduce the long-term burden on children who have had COVID-19 [60].

Smane et al. undertook a retrospective descriptive cohort study by collecting data from 92 patients under the age of 18 who presented with post-acute COVID-19 symptoms. In total, 51% of cases reported the persistent presence of at least one symptom, especially fatigue, loss of taste and/or smell, and headaches, which appeared 1 to 3 months after the onset of the disease [61].

Roge et al. included in an ambidirectional cohort study 236 patients with COVID-19, confirmed with SARS-CoV-2 infection by positive PCR test or retrospective seroconversion, and investigated the long-term repercussions, compared with 142 patients with other infections. Most COVID-19 survivors reported at least one persistent symptom, but more than half of them had multiple long-lasting symptoms, such as persistent fatigue, cognitive sequelae including irritability, mood changes, impaired attention, headache, rhinorrhea, cough, and disturbances of taste and/or smell. Long-term persistent symptoms such as fever, fatigue, rhinorrhea, loss of taste and/or smell, headache, cognitive sequelae, and night sweats have been significantly linked to COVID-19. Clinical manifestations such as persistent fatigue, cognitive sequelae, headaches, anosmia/dysgeusia, and respiratory sequelae were the most reported complaints of LC. These persistent symptoms were more evident after COVID-19 in more than half of the patients than in any other non-SARS-CoV-2 infection [62].

Molteni et al. undertook, using a mobile application, a prospective cohort study of 258,790 children volunteers (aged 5–17 years), who were reported by an adult proxy between 24 March 2020, and 22 February 2021, of whom 75,529 had valid test results for SARS-CoV-2. In total, 1734 children (588 younger and 1146 older children) were tested positive for SARS-CoV-2. Data from symptomatic children testing negative for SARS-CoV-2, matched 1:1 for age, gender, and week of testing, were also evaluated. Headache (62.2% of 1734 children) and fatigue (55% of 1734 children) as well as anosmia were the most frequent symptoms in the first 4 weeks of the disease. In only 1.8% of 1379 children, symptoms persisted for at least 56 days, and an even smaller percentage of children (15 children, 0.9%) who tested negative had symptoms for at least 28 days, but with a greater number of manifestations. After day 56, most children were symptom-free. Although COVID-19 in children is a short-term illness with mild symptoms, some children experience prolonged illness [63].

Although many publications cover the LC topic for adults, information on prevalence, pathophysiology, and clinical symptoms is not fully elucidated for children and adolescents. Even though studies show that the acute manifestations of the disease are not severe in most children with COVID-19, more and more recent clinical data highlight the important consequences of post-acute symptoms of SARS-CoV-2 infection. If we talk about COVID-19 and LC in children, the problem remains unresolved. It is still not possible to assess the frequency and the distribution of LC syndrome in pediatrics after SARS-CoV-2 infection, because the publications are heterogeneous and limited to a few countries. In children and adolescents, it is very important to distinguish LC from MIS-C, which is actually an acute manifestation with serious multiorgan and systemic involvement, high fever, and greatly altered biological parameters in a child who has tested positive or recently positive for SARS-CoV-2 infection. Currently, it is still not possible to explain how severe COVID-19 and MIS-C occur, especially in children who have come into contact with people with COVID-19. Therefore, for high-risk children and adolescents who become positive for SARS-CoV-2 following contact with patients with COVID-19, appropriate multidisciplinary management measures for several months are required [32,64,65,66].

Between October 2020 and June 2021, Trapani et al. investigated the prevalence level of long COVID-19 syndrome in a cohort of children with access to primary health care, for a study interval of 8 to 36 months after recovery and another cohort of children with access to the hospital from a number of 18 pediatric clinics covering 8 Italian regions (Liguria, Piedmont, Lombardy, Emilia-Romagna, Lazio, Puglia, Calabria, and Sardinia) with 21,663 children. The cumulative incidence of long COVID-19 symptoms was 24.3% in primary care patients and 58% in inpatients. Primary care patients had more frequent chronic fatigue and neurological and respiratory symptoms, whereas hospitalized patients had psychological manifestations, cardiac damage, and respiratory dysfunction. Regarding the prevalence of long COVID-19, it was 46.5% in symptomatic children with acute infection and 11.5% in asymptomatic ones. Young children presented more often respiratory symptoms, and adolescents neurological and psychological symptoms of long-term COVID-19. Research has confirmed that long COVID-19 is a reality in the pediatric population and that it could include patients with mild symptoms or even those without acute symptoms. The need to monitor children after acute COVID-19 and the importance of vaccination programs to prevent the consequences of the LC is revealed [67].

Miller et al. conducted a cohort study in England and Wales using data from VirusWatch, starting in mid-June 2020 via the postal service, social media, and text messages, when 53,136 people registered, until May 2021. Participants completed an online questionnaire and were followed up through weekly surveys, monthly thematic surveys, and links with the National Health Service (NHS) in hospitals and in the community about the SARS-CoV-2 testing program. A subset of approximately 11,000 participants were included in the laboratory testing sub-cohort as well as reverse transcriptase polymerase chain reaction (RT-PCR) testing of SARS-CoV-2 with nasopharyngeal swab samples. In accordance with NICE guidelines, “persistent symptoms” were defined and then coded. History of SARS-CoV-2 infection was determined using baseline self-report, data from the VirusWatch survey, and presence of SARS-CoV-2 immunoglobulin G (IgG) or digital NHS and hospital data for SARS-CoV-2. In total, 5032 children were included in the study, of which 1729 were in the sub-cohort of laboratory tests; 9.9% of children reported at least one long-term condition, of which 80.7% had a diagnosis of asthma. A total of 1062 of the children in the studied cohort had evidence of SARS-CoV-2 infection in the past or at present. The prevalence of persistent symptoms was 2.6% for children who had a history of SARS-CoV-2 infection before the onset of persistent symptoms, and 66.7% of children with persistent symptoms were among those without evidence of prior infection with SARS-CoV-2. The most common persistent symptoms were general, respiratory, and ENT. The study contributed to the database on the prevalence, risk factors, and characteristics of LC in children and signals the need for further studies and supportive measures [68].

Borch et al. published the results of a Danish national cohort study that included 37,522 children aged 0–17 years with polymerase chain reaction (PCR)-confirmed SARS-CoV-2 infection, compared to a control group of 78,037 randomly selected children who did not have a positive test result for SARS-CoV-2. An electronic survey was sent to all children from 24 March to 9 May 2021. Symptoms lasting >4 weeks were common in both groups, but they were more frequent in children with confirmed SARS-CoV-2 infection. Among preschool children, the most common symptoms were fatigue, loss of smell, and muscle weakness, whereas school children more frequently had respiratory problems, chest pain, fatigue, dizziness, muscle weakness, and loss of smell and taste. Children in the control group had more difficulty concentrating, headaches, muscle and joint pain, cough, nausea, diarrhea, and fever. In most cases, LC symptoms disappeared within 1–5 months. The authors concluded that long COVID-19 in Danish children was rare and mainly short-lived [69].

The long-term sequelae secondary to a viral infection are not new in the pathology of children and adolescents; after an infection with respiratory syncytial virus, many infants and young children remain for a long time with an excruciating cough or recurrent wheezing; furthermore, chronic fatigue syndrome together with headache and musculoskeletal and abdominal pain are characteristic symptoms in adolescents and adults after an acute episode of Epstein–Barr virus infection [70,71,72].

Kikkenborg Berg et al. conducted a national cross-sectional study among children and adolescents with a history of testing positive for SARS-CoV-2, compared to a group of matched controls with a negative test, regarding the prevalence of symptoms lasting more than 2 months and their intensity, quality, aspects of daily life, and psychological and social aspects, made through the LongCOVIDKidsDK survey.

Between 20 July 2021 and 15 September 2021, 38,152 cases and 147,212 controls were invited to participate in a survey, of which 10,997 (28.8%) cases and 33,016 (22.4%) controls responded positively to the questionnaire received. Cases in the age group 0–3 years reported at least one symptom lasting more than 2 months compared to the control group. In this age group, the most common symptoms were mood swings, rashes, abdominal pain, cough, and loss of appetite. In the group of children between 4 and 11 years of age, mood changes, problems with concentration, short-term memory loss, and skin rashes predominated, and between the ages of 12 and 14, fatigue, mood swings, concentration, or memory problems predominated. LC, defined as symptoms present for 8 weeks after a positive SARS-CoV-2 test, was present in 31.2% of 1368 children aged 0–3 years, 26.5% of 5684 children aged 4–11 years, and 32.5% of 3316 children aged 12–14 years. In conclusion, cases with a history of SARS-CoV-2 infection in all age groups reported a higher prevalence of long-lasting symptoms compared to controls. Better quality of life scores were found in older children. Many long-term symptoms were also found in the control group [73].

In a prospective cohort study, Buonsenso et al. included 507 participants from 201 households in a region of Italy with 286 children and 221 adults with PCR-confirmed SARS-CoV-2 infection to define and compare the long-term effects of this infection in children and adults. Subjects were assessed over 12 months via telephone and face-to-face visits. In total, 67% of adults had at least one persistent symptom, compared to 32% of children. In adults, females had a higher risk for persistent symptoms, but not in children. Confirmed SARS-CoV-2 infection in children had persistent symptoms in a significant proportion between 1 and 3 months, but at 6–9 months there were no more differences compared to the control group. In contrast, adults showed the same high frequency of persistent symptoms at both evaluation periods. In conclusion, children may have persistent symptoms several months after the diagnosis of acute SARS-CoV-2 infection, but less severe and less frequent than the adults they live with [74].

In another prospective cohort study on a group of 679 children, Buonsenso et al. analyzed risk factors, prevalence of persistent symptoms, and impact of variants among children with RT-PCR-confirmed SARS-CoV-2 infection. Using the ISARIC COVID-19 Health and Well-Being Follow-up Survey for Children (version 1.0 translated into Italian), subjects were assessed by telephone calls and face-to-face visits at 1–5, 6–9, and 12 or more months after diagnosis of COVID-19. In total, 488 children were detected infected during the wave of wild virus, 29 with Alpha, 42 with Delta, and 120 with Omicron variants. Persistent clinical manifestations in order of frequency included: fatigue (19%), headache (12%), insomnia (7.5%), muscle pain (6.9%), and confusion with difficulty concentrating (6.8%). Patients infected with a variant or wild-type virus had persistent symptoms and largely similar recovery rates. The convalescence period was reduced from 4% in the first 1–5 months to 0.7% at 12 months. Patterns of persistence changed depending on the variants involved at the time of infection. It is noted that a percentage of patients with COVID-19 will have long-lasting symptoms, and further studies are needed to elucidate the post-COVID state [75].

Because the impact of the family environment on the development of persistent symptoms after SARS-CoV-2 infection is less known, Haddad et al. retrospectively examined a cohort of 1267 members from 341 households with 404 children under 14 years of age, 140 adolescents aged 14–18 years, and 723 adults who had a SARS-CoV-2 infection or an exposure to SARS-CoV-2 without infection, based on three serological tests and history of infection confirmed by RT-PCR and/or serological data. Subjects answered an online questionnaire to investigate persistent symptoms one year after infection. Persistent symptoms were statistically equal between age groups in those infected, associated with the reported number of household members, but significantly more numerous in women. The authors showed that one year after infection with SARS-CoV-2, the prevalence of persistent symptoms decreased; however, adolescent girls were at particular risk of prolonged symptoms. Longer-than-usual symptoms tended to cluster within families, suggesting that research into family-level mechanisms and treatments for long-lasting COVID would be particularly valuable. The concentration of prolonged symptoms within families suggests the benefit of LC interventions at this level [76].

Güven et al. performed a retrospective study of 251 children aged 0–18 years with PCR-confirmed SARS-CoV-2 infection compared with another 251 healthy children with a negative test. Long-standing COVID symptoms, along with laboratory data and imaging tests, were assessed from hospital records. Children with COVID-19 had persistent symptoms for an average of 5 months, manifested by headache and joint and back pain. Laboratory data expressed by leukocytosis, thrombocytosis, and increased D-dimers were predictive for LC [77].

A national cohort study was conducted by Stephenson et al. on 23,048 adolescents aged 11 to 17 years who tested positive for SARS-CoV-2 compared with a pool of 27,798 PCR-negative adolescents from the Public Health England database. At 3 months post-test at study launch, a subsample completed a detailed questionnaire on demographic, physical, and psychological data, indicating fatigue (39%), headache (23.2%), and shortness of breath (23.4%) as the most common symptoms in adolescents who tested positive, compared to a lower percentage of fatigue (24.4%), headache (14.2%), and others (15.8%) in the group who tested negative. Multiple symptoms were more common in females with a positive PCR test than in the control group. Adolescents who tested positive for SARS-CoV-2 had similar symptoms to those who tested negative, but with a higher prevalence of multiple manifestations, which required a multidisciplinary intervention for mental and physical health [78].

Kuczborska et al. analyzed a questionnaire completed by the parents of 147 children (of which 70 children had the diagnosis of immunodeficiency and the remaining 77 were immunocompetent) regarding the prevalence, clinical characteristics, and vaccination status of children with immunodeficiency and COVID, compared to the control group without COVID. The results of the study showed that during the first 12 weeks post-infection, immunocompetent children were significantly more affected by long COVID than immunosuppressed ones. After this period, symptoms of chronic fatigue, reduced exercise tolerance, and difficulty concentrating and carrying out daily activities remained more prominent in the group of immunocompetent children, and immunosuppressed children complained more frequently of GI symptoms. Immunosuppressed children had significantly less intense LC symptoms compared to immunocompetent ones, an aspect that could not be explained in the authors’ study [79].

If the first reports indicated a prevalence of about 50% for children with LC, today this percentage is limited to 0.8–13.3% of children and adolescents who have been infected with SARS-CoV-2 [80].

Kostev et al. investigated in a retrospective cohort study of 6568 German children and adolescents the prevalence and factors associated with post-COVID-19 condition. The prevalence rate of the post-COVID-19 condition was estimated at 1.7% of the research group. Children over the age of 13 were more frequently affected by post-COVID-19 symptoms than those under the age of 5. Anxiety, allergic rhinitis, and somatoform manifestations predominated in the post-COVID-19 condition. In conclusion, the authors noted that the prevalence of post-COVID-19 symptoms was low in this pediatric cohort [81].

Warren et al. used data from repeated surveys in a large cohort of English schoolchildren from the COVID-19 Schools Infection Survey (SIS) for the school year 2021/22 in order to describe the weighted prevalence of post-COVID-19-condition and compare persisting symptoms between individuals with a positive SARS-CoV-2 test and those with neither a positive test history nor suspected infection. Child health data were collected in three stages—November–December 2021, January–February 2022, and March–April 2022—in a randomly stratified representative cohort. Parents and older children completed questionnaires regarding the sociodemographic composition of the household, medical history, SARS-CoV-2 test results, social contacts, and the symptoms, including mental well-being. The authors first investigated the prevalence (%) of post-COVID-19 status or LC, using the modified DELPHI consensus definition as “a history of confirmed SARS-CoV-2 infection, with at least one persisting physical symptom for a minimum duration of 12 weeks after initial testing that cannot be explained by an alternative diagnosis. The symptoms have an impact on everyday functioning, may continue or develop after … infection, and may fluctuate or relapse over time” [54]. The research results showed that, as of March 2022, a total of 7797 children in 173 schools met the conditions of post-COVID-19. Persistent manifestations of the disease for more than 12 weeks were frequently noted, especially for states of anxiety or difficulty concentrating, regardless of the previous state of infection, and they increased in percentage directly proportional to age. Loss of smell or taste, cardiovascular damage, and some systemic symptoms were detected in all age groups in the third round, more frequently in children with a previous positive test. Persistent symptoms were common in children regardless of SARS-CoV-2 test results, but loss of smell and taste were characteristic of children with a history of positive testing. The study found far-reaching effects of the COVID-19 pandemic on children’s health and well-being [82].

Jamaica Balderas et al. undertook a prospective study for the detection and management of post-COVID-19 sequelae from July 2020 to December 2021 in a cohort of 215 children aged 0–18 years who tested positive for SARS-CoV-2 by RT-PCR and/or immunoglobulin G test. Patients had neurological, endocrinological, pulmonary, oncological, and cardiological comorbidities as the most frequently observed. In total, 32.6% of children had persistent symptoms at 2 months, 9.3% at 4 months, and 2.3% at 6 months, including dyspnea, dry cough, fatigue, and runny nose. Sequelae reported as representative were alopecia, radiculopathy, perinosis, psoriasis, anxiety, and depression, with improvement at 6 months. The authors called attention to multidisciplinary and individualized management for good health and quality of life of children with persistent post-COVID-19 symptoms [83].

A cohort study of 227 participants with a mean age of 11.93 ± 2.96 years, comprising 116 children and adolescents with attention deficit hyperactivity disorder (ADHD) and 111 without ADHD, was conducted by Kara et al. with the aim of comparing the long-term effect of the COVID-19 pandemic on participation, support, and barriers at home in children with and without ADHD. Subjects with ADHD participated more actively in computer and video games compared to non-ADHD control children. The long-term impact of the COVID-19 pandemic negatively influenced participation in household activities of children with ADHD compared to those without ADHD. Cognitive demands hindered participation and engagement in the home environment, whereas the requirements relating to cognition were supportive for children without ADHD [84].

Persistent symptoms after acute COVID-19 include a variety of cognitive or neurological dysfunctions, such as loss of smell or taste, chronic fatigue syndrome, and others, known as LC after SARS-CoV-2 infection, being present in all age categories and which can limit daily activities, with a great impact on children’s development. For the accuracy of the diagnosis of LC, it is absolutely necessary to exclude other somatic or mental pathologies that have manifestations similar to the suspected disease, because LC can also trigger other diseases. Since the main groups of respiratory, gastrointestinal, neurological, psychological, and inflammatory pathologies are similar in adults and children, and since we still do not have specific biological parameters, the diagnosis is established with difficulty and delay [54,85,86,87].

Goretzki et al. investigated the rate of patients with suspected LC who initially had other illnesses to analyze whether the symptoms described in adults are similar to those in children and adolescents. A retrospective study was performed in the Outpatient Department of Pediatric Infectious Diseases of the University Hospital Essen (Germany) on a group of 110 patients with suspected LC, by correlating initial symptoms and final diagnoses with clusters of adult-onset LC. Out of the total of 110 suspected patients, 32 were diagnosed with LC according to the criteria of the German Association of Scientific Medical Societies (AWMF) [88], 21 although they met the criteria, they also had a previous illness that could probably motivate at least part of the symptoms, and the remaining 52 patients did not meet the criteria and were subsequently diagnosed with somatic or psychiatric illnesses. There was 100% agreement between the AWMF and NICE criteria administered in all patient subgroups. The positive diagnosis of LC must be finalized only by excluding other entities and only after symptomatic treatment. The use of clinical criteria and LC clusters from adults would be beneficial in early detection of patients with a low probability of this disease [89].

The need to understand LC in pediatric patients is increasingly urgent to be able to formulate specific case definitions and guidelines for an appropriate and successful management. Garai et al. included in a study 103 patients with LC syndrome, without a control group. The authors collected and analyzed the data from 89 patients who were effectively enrolled by making a diagnosis of exclusion with multidisciplinary medical examinations (physical, laboratory, and radiological examinations, specialist consultations, etc.). In total, 17 children (23%) had moderate or severe difficulties, and most patients had at least one symptom that affected their daily quality of life; in 37%, respiratory symptoms were the most frequent, and in 7% even autoimmune thyroiditis was detected. The authors concluded that evidence-based pediatric guidelines are needed in the COVID era [90].

A synthesis of published studies on LC in children and adolescents is presented in Table 1.

## 3. The Interplay between Main Molecular and Cellular Pathogenic Mechanisms in Pediatric Long COVID

Currently, for both adults and children, we still lack clear evidence about the factors involved and the pathophysiological mechanisms of the onset of LC symptoms. There are various theories and many hypotheses, but these are still limited by the heterogeneity of the available publications, so the proposed underlying mechanisms remain ambiguous. In general, it is believed that the long-lasting clinical symptoms are the result of viral aggression in the acute phase of the disease because of damage to the involved systems and organs, followed by the persistence of the SARS-CoV-2 or viral components, which, due to immune dysregulation, cannot be eliminated and which trigger autoimmunity phenomena, endothelial dysfunction, microcirculation disorders, and activation of the coagulation system [80,91].

### 3.1. Persistence of Virus or Viral Components and Prolonged Immune Activation with Inflammation

The initiation of acute SARS-CoV-2 infection is favored by the presence of the ACE2 receptors as a gateway for SARS-CoV-2. Through this gate, the virus enters and attacks the cells of the nasal cavity, oral mucosa, lungs, heart, GI tract, pancreas, liver, spleen, kidneys, brain, vascular endothelium, and others, causing damage manifested by acute symptoms, which may later become persistent or recurrent. The spike protein (S) in the structure of the coronavirus facilitates the penetration of the viral agent into the target cells. Attachment is achieved by the S1 subunit of the spike protein, which is responsible for recognizing the cell surface receptor ACE2 through a receptor binding domain (RBD), while the S2 subunit is required for membrane fusion. If the cell has fewer ACE2 receptors on its surface, the infection may be milder. Transcriptomic studies have shown that children have lower ACE2 protein expression than adults, although the extent of gene expression does not always correlate with protein levels. ACE2 receptors are encoded by a gene that can have several forms, that is, there is a polymorphism that varies from person to person. The most common polymorphisms of the angiotensin-converting receptor gene are the angiotensin-converting enzyme insertion (ACE I) and the angiotensin-converting enzyme deletion (ACE D) polymorphism [92,93,94].

For the virus to enter the cell, it is necessary to split the S protein by the proteases of the attacked cell into the S1/S2 and S2’ components. The process of fusion of virus and cell membranes is driven by the S2 subunit that binds ACE2 to be the entry receptor and uses the transmembrane protease serine 2 (TMPRSS2) to prime the S protein. A TMPRSS2 inhibitor approved for clinical use was shown to be able to prevent virus entry, an aspect that brings hope for treatment [95,96].

ACE2, in addition to being the cellular receptor for SARS-CoV-2 and other coronaviruses, plays an essential role in the regulation of inflammatory processes by decreasing the main proinflammatory peptides of the renin–angiotensin system (RAS) and the kinin–kallikrein system (KKS). These two interconnected signaling systems stimulate the inflammatory response through tumor necrosis factor alpha (TNF-α), which in turn is activated by tumor necrosis factor–alpha converting enzyme (TACE) also known as a disintegrin and metalloprotease 17 (ADAM17) protease that destroys ACE2 and recruits macrophages and neutrophils. ACE2 would have an important role in stopping this inflammatory process. If ACE2 is no longer activated or has undergone degradation, an inflammatory response will be triggered with disastrous consequences on organs, including the onset of pulmonary edema from COVID-19, but at the same time it can have long-term repercussions even after the SARS-CoV-2 infection has ceased, as observed in MIS-C. Regarding ACE2 expression in adults and children, there are conflicting opinions, but it is accepted that ACE2 expression does not change uniformly with age, starting from late fetal life, where ACE2 expression is low, and then reaching a maximum in early childhood, with large cellular and individual variations, and decline in old age. The reduction in ACE2 expression with age and the amplification of the proinflammatory components of the RAS may have a special role in supporting the inflammatory theory of the aging phenomenon and in the senescence processes. Since ACE2 is located in the X chromosome and its experimentally demonstrated expression is higher in females than in males, it may argue for the proposed negative correlation in the gender comparison. The inverse relationship between ACE2 expression and severity of COVID-19 is an exception in children, who manifest milder forms of COVID-19. This could be explained by the fact that children express less ACE2 than adults, but much more angiotensin II receptor type 2 (ATR2), which competes with angiotensin II receptor type 1 (ATR1) and blocks it, preventing its inflammatory effect. Therefore, it is believed that the high level of ATR2 receptors in children can counterbalance the low level of ACE2, and, as a result, clinical symptoms are moderate in children [97,98,99,100,101].

The extensive surface expression of the ACE2 receptors on lung alveolar epithelial cells, small intestine enterocytes, arterial and venous endothelial cells, and arterial smooth muscle cells in various organs could clarify the multitude of clinical manifestations of LC, after infection with SARS-CoV-2. Long-term effects of SARS-CoV-2 infection also occur in patients with moderate and mild forms of COVID-19, including brain fog, reduced gray matter thickness, and brain size. Research on the neuropathological characteristics of the brains of deceased patients revealed mild neuropathological changes and pronounced neuroinflammation in the brainstem. Activation of microglia and pronounced infiltration of brainstem tissue, cerebellum, and meningeal structures with cytotoxic T lymphocytes was observed in patients who died of COVID-19. Histoimmunochemical examination demonstrated the presence of SARS-CoV-2 RNA and proteins in both cranial nerves from the lower brainstem and in isolated brainstem cells from several cases who died of SARS-CoV-2 [102,103,104].

A growing body of evidence is being brought forward by researchers demonstrating that in some LC patients, the virus persists in hidden reservoirs in tissues or organs, including central nervous system structures, after acute infection [105,106].

Accumulation of SARS-CoV-2 spike protein in the skull–meninges–brain axis presents potential molecular mechanisms and therapeutic targets for neurological complications in LC patients, as a team of researchers very recently proved. Rong et al. investigated in mouse models and postmortem human tissues the presence and distribution of the SARS-CoV-2 spike protein in the skull–meninges–brain axis. The study revealed that the accumulation of spike protein in the marrow of the cranial bones and meninges for a long time after the onset of COVID-19 and then the penetration into the cerebral circulation and brain structures may participate in the emergence of the neurological manifestations of long COVID. These findings suggest that virus fragments accumulate and remain in the brain long after the virus is gone and trigger the inflammation that causes symptoms of long COVID [107].

In another study, although the presence of the virus in the brain parenchyma was not detected, the signs of an immune activation process translated by significant and persistent neuroinflammation in patients with acute COVID-19 were highlighted; it is believed that the highly immunogenic spike protein could be directly involved in the perpetuation and aggressiveness of the infection [108].

Recent publications have highlighted the presence of perivascular inflammation in the brains of patients who died of COVID-19, and others have demonstrated that spike protein could damage the vascular endothelium in an animal model and disrupt an in vitro blood–brain barrier (BBB) model, facilitating its penetration through the BBB and the occurrence of neuroinflammation. On the other hand, the spike protein seems to work together with human molecular chaperones, triggering a process of autoimmunity and the stimulation of toll-like receptors (TLR), with the release of proinflammatory cytokines. Some anti-protein antibodies cannot be neutralizing and change their conformation to bind to its receptor. Once the spike protein enters the brain or is expressed by brain cells, alone or together with inflammatory cytokines, it could stimulate microglia and initiate neuroinflammation in LC [109,110].

Prolonged or recurrent olfactory and gustatory dysfunctions are common in COVID-19, especially in patients with mild symptoms. An etiological, virological, molecular, and cellular study of the olfactory neuroepithelium in seven patients with COVID-19 who presented with acute loss of smell demonstrated the presence of viral transcripts and cells infected with SARS-CoV-2, along with phenomena of chronic local inflammation [111,112].

Neurological pathology as expressed by encephalitis, stroke, acute transverse myelitis, and cranial or peripheral nerve damage, such as Guillain-Barré syndrome, represents approximately 1% of cases reported in children. Some manifestations may occur during or after infection with SARS-CoV-2, through immune-mediated post-infectious mechanisms [113].

### 3.2. Processes Mediated by Autoantibodies in the Spectrum of Autoimmune Diseases

After the acute phase of the infection, complete removal of the SARS-CoV-2 and increased immunity will not necessarily lead to complete elimination of viral RNA, as virus- and host-related mechanisms intervene. The virus can protect itself by mutations of the genes that encode the junction or replication proteins, as well as by its ability to evade the action of the adaptive immune system. The host can defend itself by using non-cytolytic clearance mechanisms that protect against destruction of infected cells, which are specifically activated by innate immune responses in an attempt to abrogate virus replication in infected cells. Identifying the role of RNA persistence in protracted disease, how it is protected from degradation, its relative length and fragmented or defective viral genomes (DVGs), and how RNA is replicated will help us solve this mystery and possible therapeutic interventions [105].

Several months later, after infection with SARS-CoV-2, the persistence of viral messenger RNA (mRNA) and spike protein was evidenced in LC patients by positive PCR in the digestive and genitourinary tracts, by intestinal biopsy, and histopathological examination by microscopy electron and immunofluorescence. At the same time, it has been shown that the GI tract, especially in children, is a site of active viral replication of SARS-CoV-2 and can function as a long-term reservoir for this virus [114,115,116,117,118,119,120].

An immunohistochemistry and electron microscopy study of skin biopsies from seven children and adolescents with skin lesions and COVID-19 confirms vascular lesions and the presence of SARS-CoV-2 in endothelial cells. Vascular injury, inflammation, and chemotaxis persisted in patients with COVID-19 and were correlated with abnormal clinical features three months after discharge, particularly in severely recovered COVID-19 patients [121,122].

Other studies have shown that after acute infection with SARS-CoV-2, children continue to have the virus in their feces for a long time compared to adults, despite negative breath tests [123,124].

A very recent publication reported that spike fragments and/or viral RNA persist in post-COVID-19 recovered patients up to 1 year or more after acute SARS-CoV-2 infection and indicated that circulating spike protein is associated with extracellular vesicles in the absence of viral RNA inside these vesicles [125].

Extracellular vesicle (EV) is actually an umbrella name for a diversity of nano-sized particles (30–1000 nm) secreted by cells, which can consist of exosomes, cell membrane-derived microvesicles, and apoptotic bodies. EV has a lipid bilayer membrane, which gives it the ability to bind artificial ligands for various targets or incorporate microRNAs (miRNAs), messenger RNA (mRNA), DNA, lipids, biologically active compounds, or various proteins. Their value lies in the fact that they are biodegradable, biocompatible, allogeneic, and safe; therefore, they can be used as therapeutic platforms for antiviral and as anti-inflammatory and anticancer agents. The host cell infected with SARS-CoV-2 can escape the spike protein with the help of these EVs [126,127,128].

After the first 6 months of life, when the infant begins to lose the maternal protective antibodies that ensure passive immunity, he or she must face increasingly numerous external antigenic aggressions. Viral invasion of the upper airways with SARS-CoV-2 in children is counteracted by a strongly marked response through type I and type II interferon (IFN), as well as through inflammasome-dependent pathways, compared to the response mode in adults. IFN-I plays a crucial role by activating the genes that trigger the apoptosis of infected cells, the stimulation of which is earlier and more effective in children due to the multiple receptors in the cytosol, compared to the elderly, in whom this production is late, generating an insufficient tardy immune response that already allows the replication of the viral agents in the cascade. The appearance of anti-IFN autoantibodies is correlated with age; they will increase the severity of the disease by delaying the prompt reaction of the immune system against the virus, which explains why patients with severe form of COVID-19 have a high percentage of autoantibodies targeting IFN, complement, interleukins, chemokines, and other immunomodulatory proteins, including those on the cell surface. The key issues raised by the very different reactions and disease forms in COVID-19 and LC must be sought in correlation with the peculiarities of the children’s immune system, which can be a “successful immune response” compared to adults and the elderly. It was found that in small children the respiratory tract is stronger, and it defends itself much better, probably due to the training received at the frequency of viral infections and/or recent vaccinations and the changes in the local microbiome. More efficient local tissue responses, better thymic function, and reactive immunity have been proposed to explain mild clinical symptoms or even asymptomatic forms of SARS-CoV-2 infection in children [129,130,131,132].

After the COVID-19 pandemic, an increase in the number of cases of autoimmune disease is reported. The mechanism by which SARS-CoV-2 can induce these pathologies is represented by the overactivation of mature natural killer cells and CD8+ T lymphocytes, the dysregulation of B lymphocytes, and the participation of proinflammatory cytokines. It was shown that patients with COVID-19 had increased concentrations of TNF-α, interleukins IL-1 and IL-6, and leukocytes and neutrophils, whereas the number of lymphocytes, monocytes, eosinophils, and basophils was decreased [133,134].

Molecular mimicry would be one of the fundamental mechanisms by which peptide structures in the infectious agent SARS-CoV-2 similar to those of the host can activate T or B lymphocytes and trigger the phenomenon of autoimmunity, but molecular mimicry is not the only mechanism in the development of autoimmune responses. Many other factors can participate in this phenomenon, among which we mention the genetic structure of the host, the interaction with the microbiota, chemical adjuvants, disruption of immune tolerance, nonspecific activation with the persistence of some cryptic external antigens, the response of autoreactive T lymphocytes, the activation of autoimmune B cells, etc. [135,136,137].

The distorted immunological response is a key factor in the LC phenomenon in children and adolescents, as demonstrated in a recent study on 12 children with PASC (or LC) compared to 17 patients completely recovered after SARS-CoV-2 infection. In the studied group with PASC, three patients had pre-existing comorbidities (bronchial asthma, Schőlein-Henoch purpura, and Duchenne muscular dystrophy without oxygen demand)). The laboratory data objectified higher concentrations of plasmablasts, IgD-CD27+ memory, and switched IgM-IgD-B cells. Children from the control group presented significantly higher naïve and unswitched IgM+IgD+ and IgM+CD27-CD38dim B cell subsets. The serum concentrations of IL-6 and IL-1β were increased in both groups, but much higher in children with PASC, evoking the predominant participation of the innate immune system [138].

The study of IgM and IgG antibodies against the RBD of the spike protein of SARS-CoV-2 showed a significantly decreased concentration after 6 months of infection with SARS-CoV-2, whereas the number of RBD-specific memory B cells remained unchanged. This aspect can be explained by the persistence of a small amount of SARS-CoV-2 antigen or incomplete viral clearance. The hypothesis was confirmed by the presence of SARS-CoV-2 RNA and proteins in 7 of 14 intestinal biopsy samples from previously asymptomatic COVID-19 patients with a negative nasal swab PCR after a period of 4 months after acute infection [139].

Persistence of the SARS-CoV-2 or its components can induce LC symptoms by activating the host’s perverted immune response, which will trigger the generation of long-term autoantibodies that target interferon responses, type II cellular immunity, the acute phase response, leukocyte movement, and lymphocyte function or activation.

Autoantibodies directed against the endothelial adhesion molecule plasmalemma vesicle-associated protein (PLVAP), the regulator of angiogenesis r-spondin-3 (RSPO3), the metabotropic glutamate receptor, and the hypocretin receptor (orexin) receptor 2 gene responsible were detected of sleep disorder, in the central nervous system and against connective tissue, and of matrix metalloproteinases MMP7 and MMP9 [140,141].

Patients with symptoms after 3 months of acute illness more commonly show increased levels of proinflammatory cytokines (e.g., IL-1, IL-6, TNF-α), chemokines such as monocyte chemoattractant protein-1 (MCP-1) and interferon gamma-inducible protein-10 (IP-10), but also factors associated with vascular damage, such as intercellular adhesion molecule 1 (ICAM-1) and vascular cell adhesion molecule 1 (VCAM-1) [142].

Patterson et al. set out to research the presence of the SARS-CoV-2 S1 protein in a group of 144 people divided as follows: 29 normal individuals, 26 patients with mild-moderate COVID-19, 25 patients with severe COVID-19, and 64 of patients with chronic post-COVID-19 symptoms. Persistence of symptoms has been suggested to be secondary to immune stimulation by viral RNA and spike protein present in a subset of monocytes. From the initial batch, the authors investigated the presence of the SARS-CoV-2 S1 protein in 46 people. T, B lymphocyte subsets and monocytes from patients with severe COVID-19 and from those with persistent symptoms after acute infection with COVID-19 were studied. The number of intermediate (CD14+, CD16+) and nonclassical (CD14Lo, CD16+) monocytes was significantly increased in patients with PASC symptoms persisting 15 months after acute infection, compared to the control group of healthy subjects. At the same time, a significant percentage of nonclassical monocytes containing the SARS-CoV-2 S1 protein was detected in patients with severe COVID-19 but also in PASC patients 15 months after infection with SARS-CoV-2 [143].

As is known, circulating monocytes are classified into three subsets (classical, intermediate, and nonclassical) and have different functions in the immune system. Classical monocytes are involved in phagocytic activity, secrete the cytokines IL-6, IL-8, chemokine C-C motif chemokine ligand 2 (CCL2), CCL3, and CCL5, and produce large amounts of reactive oxygen species (ROS). Monocytic cells from the intermediate subset express C-C chemokine receptor type 5 (CCR5) on their surface, are antigen presenters, secrete cytokines TNF-a, IL-1β, IL-6, and chemokine CCL3 after stimulation of TLRs. The subtype of nonclassical monocytes presents on their surface high levels of CX3C motif chemokine receptor 1 (CX3CR1), participates in the phagocytosis process mediated by complement and crystallizable fragment (Fc) gamma, and is responsible for antiviral responses. After release from the bone marrow into the systemic circulation, 85% of human monocytes are of the classical subtype, and the remaining 15% are intermediate and nonclassical. The activation of nonclassical monocytes in patients with COVID-19 and the presence of S1 protein in patients with PASC indicate their role as a viral protein reservoir. In patients with PASC, high levels of the cytokine interferon-gamma (IFN-γ), which can stimulate the production of TNF-α, have been detected. High levels of TNF-α and IFN-γ induce the upregulation of the chemokine fractalkine (CX3CL1) by vascular endothelial cells, favoring the survival of nonclassical monocytes. Thus, nonclassical monocytes are perverted and acquire a proinflammatory function by activating the pathogenic nuclear factor kappa-light-chain-enhancer of activated B cells (NF-κB) pathway and stimulating the production of proinflammatory cytokines IL-1α, TNF-α, and IL-8 [144,145,146,147].

SARS-CoV-2 infection worsened psychological status, especially among children and adolescents with long-term neuropsychiatric consequences. During the acute episode, there was worsening of obsessive disorders, anxiety, restlessness, mania, anger, and tics, with decreased cognitive performance and well-being. After COVID-19, more new cases of pediatric acute-onset neuropsychiatric syndrome (PANS) and even other symptoms have emerged. In explaining this pathology, silent viral agents such as Epstein–Barr virus have been hypothesized to be involved in pathogenic mechanisms through reactivation and immune responses with chronic neuroinflammation. Epstein–Barr virus is a pathogen that is part of the human lymphotropic herpesviruses (family Herpesviridae) that can affect approximately 95% of the global population. In total, 90% of adults have been shown to have anti-EBV antibodies, evidence of previous infection. Primary EBV infection is much more common in children and adolescents, usually expressed by atypical or asymptomatic manifestations, misdiagnosed or missed. Children under four years of age, immunosuppressed, or immunodeficient may not react with specific antibodies. In other age groups, the virus sometimes cannot stimulate the body strongly enough to produce enough antibodies to be detected. If EBV-specific viral antibodies cannot be demonstrated, then DNA testing is a technique used more and more often in the clinic. PANS is a model of expression of immune-mediated neuropsychiatric symptoms, as reactivation of a latent virus and a prototype of chronic post-acute manifestations of COVID-19. EBV reactivation can occur after infection with SARS-CoV-2 or concurrently with COVID-19, including initially asymptomatic patients. SARS-CoV-2 can train other infectious agents (e.g., cytomegalovirus) to participate in the long-term symptoms of COVID. More and more studies claim that long-term symptoms of COVID are a consequence of inflammation caused by SARS-CoV-2 or its components through reactivation of EBV. SARS-CoV-2 can stimulate subclinical chronic inflammation. Chronic recurrent/reactivated EBV infections are accompanied by the onset of several autoimmune diseases, including diabetes. If chronic or recurrent EBV infection affects epithelial cells, systemic lupus erythematosus and Sjögren’s syndrome can occur, and when B cells are infected, chronic arthritis, multiple sclerosis, and other diseases can occur. These secondary autoimmune diseases present as overlapping syndromes with clinical and biological manifestations expressed by specific autoantibodies (e.g., antinuclear antibodies and rheumatoid factors) that reflect epithelial and/or B-cell infection [148,149,150,151,152].

Other secondary manifestations of SARS-CoV-2 infection are those that affect the muscular system and the locomotor apparatus present in a percentage of 10–25% of patients with COVID-19. Suffering is manifested by asthenia, myalgia, joint pain, back pain, etc. Myalgia, protracted myositis, rhabdomyolysis, and “autoimmune necrotizing myositis” induced by SARS-CoV-2 have been reported by many publications in patients with COVID-19. During infection, release of proinflammatory cytokines with myotoxic potential, deposition of immune complexes, and damage caused by similarities between viral antigens and human muscle cells are considered immune-mediated mechanisms of muscle damage. Cachexia and sarcopenia may occur as muscle sequelae of long-term post-COVID-19 evolution. Reduction in muscle mass by destruction of fibers and infiltration with adipose tissue are detected by magnetic resonance imaging (MRI) in the case of muscle atrophy from cachexia and sarcopenia [153,154,155].

During the COVID-19 pandemic, there has been a sharp increase in new cases of juvenile dermatomyositis (JDM) in older female children. Some infectious agents such as parvovirus B19, coxsackie virus B, enterovirus, influenza, group A streptococcus, and toxoplasma gondii are known to trigger seasonal JDM. At the same time, infection with SARS-CoV-2 can be a strong trigger for JDM. It seems that dysregulation of the type I IFNs signaling pathway can lead to the expression of myxovirus-resistance protein A with its deposition in capillaries and muscle fibers and could be one of the hypotheses in the pathogenesis of JDM. Prolonged post-viral myositis documented by MRI occurs following SARS-CoV-2 infection approximately 3–7 days with diffuse or multifocal muscle pain and/or rhabdomyolysis and may last much longer, which is another hypothesis. This last entity is a syndrome of hyperinflammation such as MIS-C, KD or dermatomyositis, but it is not true dermatomyositis [156,157,158,159].

In addition to dermatomyositis, other entities such as juvenile idiopathic arthritis, reactive arthritis, spondyloarthritis, psoriatic arthritis, and systemic lupus erythematosus are part of the chronic rheumatic diseases that could start after SARS-CoV-2 infection [160,161,162,163,164,165,166,167].

In recently published studies, it is mentioned the particularly high risk for patients who have had COVID-19 of developing after a few months autoimmune diseases such as systemic sclerosis, mixed diseases of connective tissue disease, Behçet’s disease, polymyalgic vasculitis, inflammatory bowel disease, celiac disease, type 1 diabetes, etc. Attention is drawn to the link between COVID-19 and the risk of these autoimmune diseases, so that they are recognized in time and patients can receive early treatment [137,168,169].

### 3.3. Long COVID in the Era of the Microbiome and Connected Molecular Mechanisms

Research and scientific knowledge about SARS-CoV-2 infection and its consequences on the human body developed gradually as the pandemic unfolded. Although the primary site of infection is in the respiratory system, SARS-CoV-2 can be found in the GI tract and has been isolated from stool samples with evidence of virus activity in the GI. The virus was detected in the esophagus, stomach, duodenum, and rectum and in fecal samples from COVID-19 patients. Long-term GI manifestations, especially diarrhea, have been linked to decreased richness and diversity of gut microbiota, immune deficiencies, and delayed virus clearance. Operating in two directions, the interactions between the respiratory mucosa and the gut microbiota, on the gut–lung axis, are supposed to be involved in the immune responses to infection. As in other respiratory infections, gut dysbiosis is linked to heightened inflammation and diminished regulatory or anti-inflammatory processes in both the lung and gut, highlighting this significant connection between the two mucosal compartments. Investigations have pointed to the occurrence of intestinal dysbiosis in infected patients, with exacerbated inflammation and decreased regulatory or anti-inflammatory mechanisms, as a possible interrelationship in terms of disease severity. An open question that has been raised is whether dysbiosis is a consequence or a contributing cause of the severity of SARS-CoV-2 infection, such that treatments aimed at decreasing GI permeability may be salutary [168,170,171].

The damage outside the lungs triggered by COVID-19 includes the gut, because the virus can remain present where ACE2 receptors are strongly represented. GI manifestations involve diarrhea, vomiting, and abdominal pain existing in many patients due to the tropism of SARS-CoV-2 for ACE2. Gut homeostasis and the microenvironment play a vital part in ameliorating systemic inflammation. There is still insufficient information on residual GI symptoms of SARS-CoV-2 infection. This virus clearly and indirectly affects gut physiology in various ways; consequently, different functional disorders of the gut will take place after recovery, such as dysbiosis, disruption of the intestinal barrier, microinflammation of mucosa, post-infectious conditions, immune abnormalities in the regulation of metabolism, and physiological or even psychological stress due to possible pathophysiological changes [172].

Diet, environmental factors, and genetics all participate as important players in shaping the gut microbiota that influence immunity [173].

It is known that among environmental factors, the microbiome modulates immune responses to infection. The boundary that separates the host tissue from this microbial community is constituted by the intestinal epithelium and the mucus layers, where immune cells continuously pick up antigens from the lumen, as well as from the destroyed mucosal barrier triggered by infection, the physical damage or inflammation of disturbed tissues, which facilitates direct interplay between host immune cells and the microbial congregation. Immunocompromised patients have a “leaky” gut due to chemotherapy or opportunistic infections, leading to important microbial translocation that precipitates abnormal proinflammatory immune cell responses. The mechanisms of the evolutionary origin of variants of concern for SAR-CoV-2 are still a matter of debate. Chronic infections with SARS-CoV-2 in certain people who are immunosuppressed is one of the hypotheses. For immunocompromised children and adolescents, permanent surveillance of the possible impact of variant strains is necessary, and vaccination must be carefully considered. [172,174,175,176,177,178].

The role of immunomodulation in the treatment of LC is more and more obvious. Clinical symptoms in LC are modulated by proinflammatory and effector physical and biochemical characteristics, as determined by both genetic makeup and environmental influences induced by SARS-CoV-2 infection. Evaluation of cytokines and lymphocyte subsets at a long distance of at least 7 months from acute infection with SARS-CoV-2 shows that latent autoimmunity and not hidden autoimmunity continued firmly throughout this time. Proinflammatory phenomena were highlighted by the increase in interleukins IL-1β, IL-6, IL-13, and IL-17A, as well as the concentrations of IFN-α, TNF-α, and granulocyte colony-stimulating factor (G-CSF), simultaneously with the reduction in IP-10, induced by interferon-γ. Many effector T lymphocytes, CD8+, T effector CD4+ with memory, and naive B cells were recorded in LC. Total IgG S1 anti-SARS-CoV-2 antibodies levels remained high over time, so that this long-term remaining immune activation can concur to the progress of latent or expressed autoimmunity. Chronic inflammatory processes with increased numbers of B cells, as primary source of autoantibodies, support autoimmune mechanisms in LC [179,180,181].

There is less known regarding the degree of gut microbiome dysbiosis in children relating to disease severity during COVID-19 infection. Higher frequency of asymptomatic cases in children, as well as the symptomatic forms of COVID-19, represents a very interesting open direction of research regarding microbiome differences, by comparison with adults. LC-associated gut microbiome dysbiosis revealed higher levels of *Ruminococcus gnavus* and *Bacteroides vulgatus* as well as lower levels of *Faecalibacterium prausnitzii* (*F. prausnitzii*) in adults. Analysis of stool in asymptomatic infants with COVID-19 revealed a decrease in *Bifidobacterium bifidum*, *Akkermansia muciniphila*, *Eubacterium limosum*, *Enterocloster clostridioformis*, *Blautia hominis*, *Veillonella dispar*, and *Enterobacter cloacae*, of which the first two are of particular importance as they are known for anti-inflammatory bacterial taxa. Bifidobacterium, as one of the most investigated for its positive effects could be used to modulate the intestinal TJ barrier and to protect and treat gut inflammation [182].

There is an urgent need to search and develop innovative treatments to successfully interact with the human microbiota and immune system in the coronavirus crisis. New solutions, molecules, and probiotics should be designed and investigated to be innovatively applied to discover how bacteria can help us fight this pandemic, so that we can find the key to the hidden code of communication between RNA viruses, bacteria, and our body [183].

**Figure 2:**
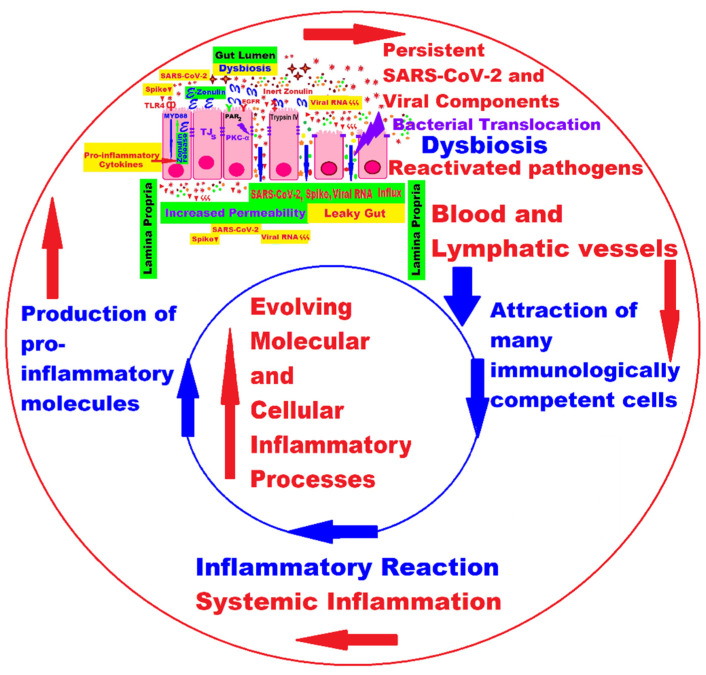
Persistence of SARS-CoV-2 and its remnants, intestinal dysbiosis, bacterial translocation, reactivation of pathogens, and their influence on the evolution of molecular and cellular processes in LC [168].

Intestinal inflammation and gut barrier dysfunction as well as microbial translocation trigger changes in the gut microbiome. SARS-CoV-2 in the GI tract results in the release of zonulin, an important coordinator of intercellular tight junctions (TJs) between epithelial cells; higher levels of zonulin increase intestinal permeability (the “leaky gut”), which in turn facilitates trafficking of SARS-CoV-2 antigens in the blood and lymph flow, leading to hyperinflammation and autoimmune phenomena [168,184,185], as shown in Figure 2.

After acute infection with the SARS-CoV-2 associated with significant lung damage, there is a percentage of about 22% of patients who remain with GI symptoms, fatigue, sleep disturbances, mood disturbances, and cognitive dysfunction (“brain fog” and impaired memory) post-COVID-19. The mechanisms by which prolonged GI manifestations and neuropsychiatric symptoms occur in former patients with COVID-19 are unclear. From the data in the specialized literature, it can be observed that the modified structure of the intestinal microbiome, especially the reduction in butyrate and the increase in proinflammatory activities, are some of the causes of these disorders. In patients with chronic COVID-19, reduction in commensal bacteria with immunomodulatory power was observed, including *F. prausnitzii*, *Eubacterium rectale*, and *Bifidobacteria* strains; at the same time, a significant increase in proinflammatory taxa (for example, *Prevotella* and *Veillonella*) was detected in the oral microbiome [186,187,188,189].

Infection with the SARS-CoV-2 could alter certain microbial communities that disrupt the release of beneficial bacterial metabolites, subsequently inducing neurogenesis or neuroinflammation in central nervous system structures that facilitate the emergence of cognitive dysfunctions in LC. Persistent GI symptoms and chronic neuropsychiatric manifestations together with prolonged gut dysbiosis suggest impairment of the post-infectious microbiota–gut–brain (MGB) axis that could elucidate the cause of these symptoms in post-COVID-19 patients. Perturbation of the MGB axis of bidirectional communication between the intestinal environment and the brain can lead to various symptoms or diseases, including anxiety, cognitive deficits, and depression. Altered gut microbial colonization has been shown to be associated with behavioral disturbances in patients with irritable bowel syndrome, inflammatory bowel disease, metabolic disease (obesity, diabetes), autism in children, and neurodegenerative diseases (Parkinson, Alzheimer’s) in adults. Original, smart, and targeted multifunctional solutions and new nanodrugs may soon be developed to treat, for example, Alzheimer’s disease by targeting TJs not only at the brain level, but also at the gut level, with possible applications in long COVID in children, too [190,191,192].

Neuroinvasion of the brain by SARS-CoV-2 following intestinal infection and the zonulin hypothesis was highlighted. Zonulin, as a protein with an important role in the reversible regulation of epithelial permeability, released in large quantities in the intestinal lumen, can influence the integrity of TJs and the continuous efflux of antigens to the submucosa, generating inflammatory processes and severe immune reactions, with an effect on the severity of SARS-CoV-2 infection and triggering systemic inflammation. Among the possible pathways of neuroinvasion by SARS-CoV-2, an alternative pathway after gut infection, the zonulin hypothesis has been proposed, involving toll-like receptor 4 (TLR4), zonulin, protease-activated receptor 2 (PAR2), and the brain receptor zonulin [168,193,194].

Neurological and neuropsychological symptoms (fatigue, headaches, cognitive problems, and mood swings) post-COVID-19 are heterogeneous, but there is a similarity of brain patterns between children and adults, as can be seen from data published in the scientific literature. These manifestations could be explained by the hypometabolism of certain areas of the brain in long-term COVID-19. On the other hand, the dysfunction of the hypothalamic–pituitary–adrenal axis, modulated by the unbalanced gut microbiota, foreshadows that disturbed stress reactions may underlie the neuropsychiatric symptoms in LC. In pediatric pathology, neurological symptoms tend to decrease over time, and psychological symptoms remain present in large numbers for a long time in patients aged 6–17 years [195,196,197,198].

It is not yet known exactly whether the neuropsychiatric symptoms observed in children and adolescents with long COVID are the direct result of the infection or are due also to the stress caused by the restrictive measures during the pandemic and the illness itself. The neuroinflammation and the response molecules generated in the brain due to the persistence of the virus and its components could lead to an avalanche of phenomena that command the immune cells in the intestine, which should normally fight pathogens, but end up causing inflammation in the gut. Psychological assistance should be an important part in managing long COVID in children and adolescents [122,199,200].

## 4. Final Remarks and Conclusions

While the COVID-19 pandemic has caused great suffering to adults around the world, two clinical entities after infection with SARS-CoV-2 have emerged in children and posed a challenge to pediatricians: MIS-C and long COVID. If the first really endangered their lives due to the sometimes fulminant evolution, the second is still unsolved and raises many questions of diagnosis, treatment, and outcome.

LC is a multisystemic condition that affects children of all ages. Children may have persistent symptoms several weeks after the diagnosis of acute SARS-CoV-2 infection, but less severe and less frequent than the adults, and female children may be more affected.

Young children show more frequent respiratory symptoms, whereas adolescents have long-term neurological and psychological manifestations. Adolescents who tested positive for SARS-CoV-2 had symptoms similar to those who tested negative, but with a higher prevalence of multiple manifestations, which requires multidisciplinary mental and physical health interventions.

The need to easily recognize LC in pediatric patients is increasingly urgent to be able to formulate specific case definitions and guidelines for appropriate and successful management.

Scientific research supports a better understanding of LC, the need for monitoring children and adolescents after acute COVID-19, and the value of vaccination programs to prevent LC.

Why some children are at greater risk of contracting SARS-CoV-2 infection than others, why symptoms may vary among infected children, why some children have prolonged illness, and how to identify children at risk of severe disease, including MIS-C or LC, are still open questions without well-defined answers. Immunosuppressed children show significantly less intense LC symptoms compared to immunocompetent ones, an aspect that has not yet been explained. The concentration of prolonged symptoms within families suggests the benefit of LC interventions at this level.

**Figure 3:**
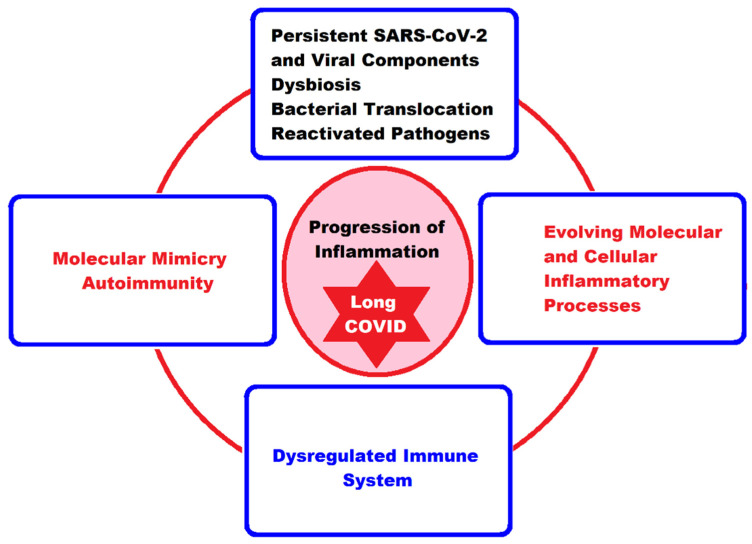
The vicious cycle and the progression of inflammatory processes in LC.

The long duration of clinical symptoms in LC could be the consequence of viral aggression during the acute period of the COVID-19, when damage to systems and organs occurred, followed by the persistence of SARS-CoV-2, its viral components, dysbiosis, bacterial translocation, and/or the reactivation of pathogens, which could not be eliminated by the deregulated immune system, and through molecular mimicry can trigger autoimmunity phenomena, endothelial dysfunction, microcirculation disorders, and so on. The schematic representation of the vicious cycle leading to chronic inflammation and LC is depicted in Figure 3.

After the virus has been eliminated, its fragments may still remain hidden in various organs, which accumulate especially in the gut and brain and can trigger the chronic inflammation that causes the symptoms of LC.

There is an important connection between the mucous compartments of the lungs and the intestine. The appearance of intestinal dysbiosis in patients infected with SARS-CoV-2 with exacerbated inflammation by deregulated and low anti-inflammatory mechanisms can modulate the severity of the disease by virtue of this interrelationship on the lung-intestine axis.

The microbiome modulates immune responses to infection because immune cells continuously pick up antigens from the intestinal lumen, but also from the mucosal barrier that is destroyed by infection, physical aggression, and inflammation of disturbed tissues, which will facilitate a direct interplay between host immune cells and the microbial congregation.

SARS-CoV-2 attaches to ACE-2 receptors in the gut through the spike protein. Following this process, an inflammatory response occurs with the strong release of proinflammatory cytokines, simultaneously with the attraction and activation of elements of the innate immune system, together with the response of the adaptive immune system. In the event of dysbiosis, there is an erosion of the mucosal barrier and other elements of the gut protective barrier, the reduction in commensal bacteria, and as a result the permeability for pathogens increases and also microbial translocation, phenomena that stimulate even more strongly the proinflammatory processes, which potentiates the virus’s ability to attack, realizing a vicious inflammatory circle.

**Figure 4:**
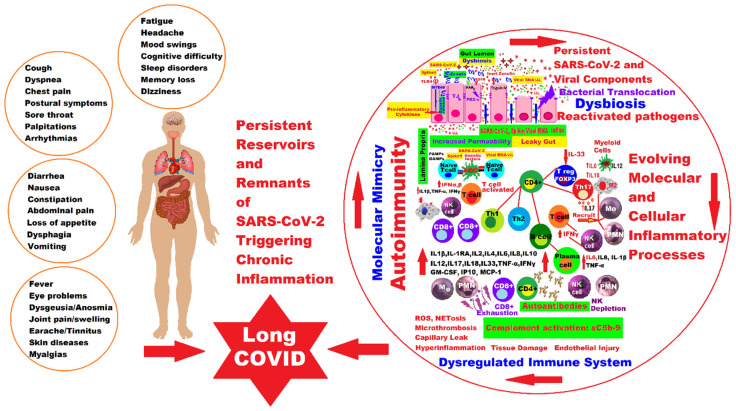
The entire complex interplay between infection, dysbiosis and inflammation in pediatric long COVID. The central pathways were drawn by the main author, L.M.A. [168].

A synthesis of all the molecular and cellular mechanisms correlated with the clinical symptoms of LC in children and adolescents presented and discussed in this work is highlighted in Figure 4.

Future discoveries and innovative ideas regarding the interplay between infection, dysbiosis, and inflammation in long COVID will help shape future pandemics, influencing clinical practice and paving the way for innovative treatments in the fine-tuning of innate and adaptive immune mechanisms.

Basic questions about LC frequency in CYP and its effects on vaccination, reinfection, post-infectious viral persistence, neuroinflammation, redundant blood coagulation and immune imbalance, mimicry, permanent tissue damage, and autoimmunity triggered by the latest variants of SARS-CoV-2 are still open.

## Figures and Tables

**Table 1:** Long COVID symptoms and recovery from SARS-CoV-2 infection in children and adolescents.

References	Type of Study	Symptoms							Conclusions
		Pulmonary	Cardio-Vascular	Gastro-Intestinal	Neurologic	Mental	Skin	Others	
[59] (Ludvigsson, J.F., 2021)	Case report and systematic review	+	+	+	+	+	+	+	**Children could have LC manifestations just like adults.**
[60] (Buonsenso, D. et al., 2021)	Cohort study on 129 children diagnosed with COVID-19	+	+	+	+	+	+	+	**Decision makers and pediatricians must get involved to reduce the long-term effects of COVID-19 in children.**
[61] (Smane, L. et al., 2021)	Retrospective descriptive cohort study; 92 patients (age ≤18 yrs)	+	+	+	+	+	+	+	**More than half of the children studied presented 1–3 months after the acute episode mainly fatigue, loss of taste/smell, and headaches.**
[62] (Roge, I. et al., 2021)	Ambidirectional cohort study. 236 patients after COVID-19 compared to 142 children with other infections.	+	-	-	+	+	-	-	**Persistent symptoms were more evident after COVID-19 than in any other non-SARS-CoV-2 infection.**
[63] (Molteni, E. et al., 2021)	Prospective cohort study using a mobile application.	-	-	-	+	+	-	+	**A holistic modus operandi is indicated as appropriate for all children with persistent illness during this COVID-19 pandemic.**
[67] (Trapani, G. et al., 2022)	Study on two cohorts that included a number of 21,663 children.	+	+	-	+	+	+	+	**Children should be monitored after acute infection with SARS-CoV-2 to avoid LC, and the vaccination programs are necessary.**
[68] (Miller, F. et al., 2022)	Cohort study in England and Wales including 5032 children with history of SARS-CoV-2 infection.	+	+	+	+	+	+	+	**Expanded the database on the prevalence, risk factors, and characteristics of LC in children. It remains unclear to what extent children are affected by persistent SARS-CoV-2-related symptoms.**
[69] (Borch, L. et al., 2022)	Danish national cohort study of 37,522 children with SARS-CoV-2 infection, RT-PCR positive, compared to a control group of 78,037 randomly divided children with a negative test for SARS-CoV-2.	+	-	+	+	+	-	+	**Long COVID in Danish children was uncommon and mainly of short duration.**
[73] (Kikkenborg Berg, S. et al., 2022)	Nationwide cross-sectional Danish study for children with a confirmed SARS-CoV-2-positive RT-PCR test and matched controls.	+	-	+	+	+	+	+	**In all age groups, cases with a history of SARS-CoV-2 infection had a higher prevalence of LC symptoms compared to controls.**
[74] (Buonsenso, D. et al., 2022) https://doi.org/10.3389/fped.2022.834875	Prospective cohort study in 286 children and 221 adults with RT-PCR confirmed SARS-CoV-2 infection.	+	+	+	+	+	+	+	**Children presented with persistent multisystemic symptoms several months after diagnosis of mild acute SARS-CoV-2 infection, although less frequently and less severely than cohabiting adults. Urgent need for further studies.**
[75] (Buonsenso, D. et al., 2022) https://doi.org/10.3390/jcm11226772	Prospective cohort study on 679 children with COVID-19 variants.	+	+	+	+	+	+	+	**Children develop long-lasting persistent symptoms and further studies are needed.**
[76] (Haddad, A. et al., 2022)	Retrospective controlled, multicenter study cohort of 1267 members from 341 households with 404 children under 14 years of age, 140 adolescents, and 723 adults.	+	+	+	+	+	+	+	**Persistent symptoms clustered within families, so family-level interventions could be useful for LC.**
[77] (Güven, D. et al., 2022)	Retrospective study of 251 children SARS-CoV-2 confirmed by RT-PCR, compared to a control group.	-	-	-	+	+	-	+	**Functional impairment between** **1 and 9 months after the onset of the infection.** **Authors indicated the most powerful laboratory predictors for LC.**
[78] (Stephenson, T. et al., 2022)	National cohort study on 23,048 adolescents who tested positive for SARS-CoV-2, compared with a pool of 27,798 PCR-negative adolescents.	-	-	-	+	+	+	+	**LC requires assessment of mental as well as physical health. At 3 months post-test, the study group had a higher frequency of all symptoms, especially multiple physical symptoms, compared to controls.**
[79] (Kuczborska, K. et al., 2022)	Two cohorts of children with immunodeficiency (n = 70), and the control group (n = 77), immunocompetent children.	+	+	+	+	+	+	+	**Immunosuppressed children had significantly less intense LC symptoms compared to immunocompetent ones, an aspect that could not be explained in the authors’ study.**
[81] (Kostev, K. et al., 2022)	Retrospective cohort study of 6568 children and adolescents.	-	+	+	+	+	+	+	**Post-COVID-19 condition was rare in COVID-19 children and adolescents in Germany.**
[82] Warren-Gash, C. et al., 2023)	Randomly stratified representative cohort of 7797 children (from 173 schools), who met the conditions of post-COVID-19	+	+	+	+	+	+	+	**Wide-ranging impacts of the COVID-19 pandemic on the health and wellbeing of CYP.**
[83] (Jamaica Balderas, L.M.D.C. et al., 2023)	Prospective cohort study of 215 children tested positive for SARS-CoV-2.	-	-	+	+	+	+	+	**Significant clinical improvement 6 months after the onset of infection. The need for a multidisciplinary and individualized approach.**
[84] (Kara, O.K. et al., 2023)	227 children, as follows: 116 children with ADHD and 111 children without ADHD.	-	-	-	+	+	-	+	**Children with ADHD did not participate in activities at home, played more video games, and were less involved in preparing school activities and homework than their healthy peers, while cognitive demands were a support for non-ADHD children.**
[89] (Goretzki, S.C. et al., 2023)	Retrospective study of 110 pediatric patients with suspected LC	+	+	+	+	+	+	+	**The use of clinical criteria and clusters of LC from adults would be beneficial in early detection of patients with a low probability of LC.**
[90] Garai, R. et al., 2023)	89 children diagnosed with LC syndrome. Without control group.	+	+	-	+	+	+	+	**Most of the children reported at least one persistent symptom that affected their quality of life. Controlled studies are needed.**

Legend: “+” = present symptom; “-“ = absent symptom.

## Data Availability

The literature used for this article is available from the first author.

## Abbreviations

ACE-2	Angiotensin-converting enzyme 2
ADAM17	A disintegrin and metalloprotease 17
ATR1	Angiotensin II receptor type 1
ATR2	Angiotensin II receptor type 2
ACE D	Angiotensin-converting enzyme deletion
ACE I	Angiotensin-converting enzyme insertion
APC	Antigen presenting cells
ADHD	Attention deficit hyperactivity disorder
BBB	Blood–brain barrier
CDC	Centers for Disease Control and Prevention
CCL2	Chemokine C-C motif chemokine ligand 2
CCL3	Chemokine C-C motif chemokine ligand 3
CCL5	Chemokine C-C motif chemokine ligand 5
CX3CL1	Chemokine fractalkine
CCR5	C-C chemokine receptor type 5
CYP	Children and young people
COVID-19	Coronavirus disease 2019
CCS	Chronic COVID-19 syndrome
CX3CR1	CX3C motif chemokine receptor 1
CS	Cytokine storm
DAMP	Damage-associated molecular pattern
DVG	Defective viral genome
DNA	Deoxyribonucleic acid
EDs	Emergency departments
EV	Extracellular vesicle
*F. prausnitzii*	*Faecalibacterium prausnitzii*
Fc	Fragment crystallizable
GI	Gastrointestinal
AWMF	German Association of Scientific Medical Societies
IS	Immune system
IgA	Immunoglobulin A
IgG	Immunoglobulin G
IgM	Immunoglobulin M
ICAM-1	Intercellular adhesion molecule 1
IFN	Interferon
IP-10	Interferon gamma-inducible protein-10
IFN-γ	Interferon-gamma
IL-1, IL-6, IL-8, IL-15	Interleukin 1, 6, 8, 15
IL-1α	Interleukin 1α
IL-1β	Interleukin 1β
IHR	International Health Regulations
IEL	Intraepithelial CD8+ lymphocytes
JDM	Juvenile dermatomyositis
KD	Kawasaki disease
KKS	Kinin–kallikrein system
LC	Long COVID
MRI	Magnetic resonance imaging
MMP	Matrix metalloproteinase
MMP7	Matrix metalloproteinase 7
MMP9	Matrix metalloproteinase 9
MERS	Middle East respiratory syndrome
mRNA	Messenger RNA
MGB	Microbiota-gut-brain
miRNAs	MicroRNAs
MERS	Middle East respiratory syndrome
MCP-1	Monocyte chemoattractant protein-1
MIS-C	Multisystem inflammatory syndrome in children
MIS-N	Multisystem inflammatory syndrome in neonates
MYD88	Myeloid differentiation primary response 88
NHS	National Health Service
NICE	National Institute for Health and Care Excellence
NIHR	National Institute for Health Research
NIH	National Institutes of Health
NF-κB	Nuclear factor kappa-light-chain-enhancer of activated B cells
PAMP	Pathogen-associated molecular pattern
PANS	Pediatric acute-onset neuropsychiatric syndrome
PIMS-TS	Pediatric Multisystem Inflammatory Syndrome, Temporarily Associated with SARS-CoV-2
PLVAP	Plasmalemma vesicle-associated protein
PCR	Polymerase chain reaction
PC	Post COVID syndrome
PCC	Post COVID-19 condition
PASC	Post-acute sequelae of SARS-CoV-2 infection
ROS	Reactive oxygen species
RBD	Receptor binding domain
RSPO3	Regulator of angiogenesis r-spondin-3
Tregs	Regulatory T lymphocytes
RAS	Renin–angiotensin system
RT-PCR	Reverse transcriptase polymerase chain reaction
RNA	Ribonucleic acid
SARS	Severe acute respiratory syndrome
SARS-CoV-2	Severe acute respiratory syndrome coronavirus 2
S	Spike protein
Th1	T-helper 1 or T helper type 1
Th2	T-helper 2 or T helper type 2
Th17	T-helper 17 or T helper type 17
TJs	Tight junctions
TLR2	Toll-like receptor 2
TLR4	Toll-like receptor 4
TLR	Toll-like receptors
TMPRSS2	Transmembrane serine protease 2
TMPRSS4	Transmembrane serine protease 4
TNF-α	Tumor necrosis factor alpha
TACE	Tumor necrosis factor-alpha converting enzyme
US	United States
FDA	U.S. Food and Drug Administration
UK	United Kingdom
UKRI	UK Research and Innovation
VCAM-1	Vascular cell adhesion molecule 1
WHO	World Health Organization
Increased	↑
Decreased	↓
Present	+
Absent/Missing	-
